# Transcriptomic data during development of a two-spotted cricket *Gryllus bimaculatus*

**DOI:** 10.1016/j.dib.2021.107388

**Published:** 2021-09-20

**Authors:** Nobuaki Kono, Hiroyuki Nakamura, Rintaro Ohtoshi, Kazuharu Arakawa

**Affiliations:** a Institute for Advance Biosciences, Keio University, Tsuruoka, Yamagata 997-0017, Japan; b Faculty of Environment and Information Studies, Keio University, Fujisawa, Kanagawa 252-0882, Japan; c Systems Biology Program, Graduate School of Media and Governance, Keio University, Fujisawa, Kanagawa 252-0882, Japan; d Spiber Inc., Tsuruoka, Yamagata, 997-0052, Japan

**Keywords:** Transcriptome, Cricket, Gryllus bimaculatus, Development, Instar

## Abstract

The two-spotted cricket *Gryllus bimaculatus* is a popular food for reptiles and other insectivorous animals, for the ease of breeding and rich nutrients. It goes through eight moulting cycles until it grows into an adult of size around 30–40 mm, but different larval instars are also used for their sizes matching the fed animals. We therefore provide a transcriptomic resource on different developmental stages of *G. bimaculatus* to understand the inner molecular workings of these stages contributing to varying nutrients. The raw RNA sequence data is available at NCBI Sequence Read Archive (SRA) under the BioProject PRJNA716138 and the assembled contigs are available as a supplementary data of this report.

## Specifications Table

**Table:** 

Subject	Entomology and insect science
Specific subject area	Transcriptomics
Type of data	RNA-seq data (paired-end) and assembly of reads
How data were acquired	Illumina NextSeq 500 sequencing platform
Data format	Raw sequence reads (FASTQ), assembled contigs (FASTA), and expression quantification (Tab delimited text of TPM values)
Parameters for data collection	1st instar, 2nd instar, 3rd instar were purchased from Tsukiyono Farm, Japan (https://tsukiyonofarm.jp), and wingless juvenile of size 6–10 mm (size S), wingless juvenile of size 10–15 mm (size S), wingless juvenile of size 15–20 mm (size ML), and winged adult female of *Gryllus bimaculatus* purchased from Mito-Korogi, Japan. 1st instar, 2nd instar, 3rd instar stages were based on 1st, 2nd, and 3rd week after hatching for the molting cycle takes about a week in these stages, and they were further confirmed by the body sizes of 2.5–3.5 mm, 3.5–5.0 mm, and 5.0–8.0 mm in these stages, respectively.
Description of data collection	Total RNA was extracted and purified from the whole body of a cricket specimen, which was sequenced as paired-end reads. Reference assembly was produced by merging reads from all conditions and subsampling 6 M paired reads, using Bridger software.
Data source location	Institution: Institute for Advanced Biosciences, Keio University City/Town/Region: Tsuruoka/Yamagata Country: Japan
Data accessibility	Repository name: NCBI SRA Data identification number: PRJNA716138 Direct URL to data: http://www.ncbi.nlm.nih.gov/bioproject/716138 Instructions for accessing these data:

## Value of the Data

• The transcriptome data for *G. bimaculatus* taken in multiple developmental stages would facilitate developmental study of arthropods to study the comprehensive gene expression dynamics through these life stages.
• Entomologists studying insect development, as well as researchers rearing insectivorous animals that are fed with different developmental stages of crickets can use this data to explore the expression changes between the stages, and to gain insights into the varying nutrients produced.
• Due to the ease of breeding, crickets are also gaining attention as a possible alternative protein source for animal-free meat. Transcriptome data for different developmental stages will allow these applications of cricket as food source by elucidating the differential metabolism and biosynthesis of various nutrients produced.

## 1 Data Description

The dataset contains raw RNA-Seq data obtained from seven life stage samples of *G. bimaculatus*, namely, 1st instar, 2nd instar, 3rd instar were purchased from Tsukiyono Farm, Japan (https://tsukiyonofarm.jp), and wingless juvenile of size 6–10 mm (size S), wingless juvenile of size 10–15 mm (size S), wingless juvenile of size 15–20 mm (size ML), and winged adult female, purchased from Mito-Korogi, Japan (http://www2u.biglobe.ne.jp/∼m-korogi/). Summary of raw read data is shown in Table 1. 12–18 M paired reads were obtained, where more than 80% of the reads had average quality value above Q30. The raw RNA sequence data is available at NCBI Sequence Read Archive (SRA) under BioProject PRJNA716138.

**Table 1:** Summary of RNA-Seq data.

	Number of paired reads	Percentage of average Q30 reads
1st Instar	17,461,709	0.838
2nd Instar	12,816,612	0.836
3rd Instar	18,664,030	0.834
Size S (6–10 mm)	15,400,493	0.818
Size M (10–15 mm)	15,666,493	0.822
Size ML (15–20 mm)	17,732,526	0.820
Adult	16,203,900	0.832

**Fig. 1:**
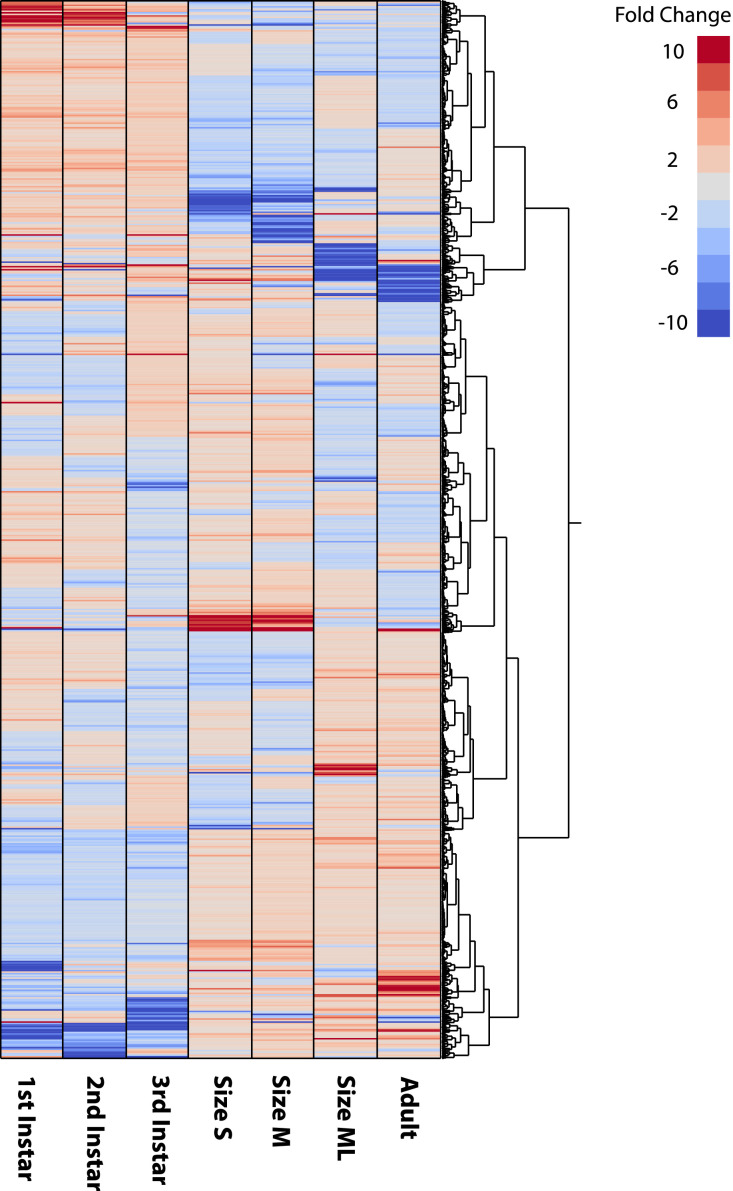
Hierarchical clustering of normalized TPM values (fold change relative to median expression level for each gene) for gene with maximum TPM > 5 and minimum TPM > 0. The expression profile shows development stage-specific expression.

**Fig. 2:**
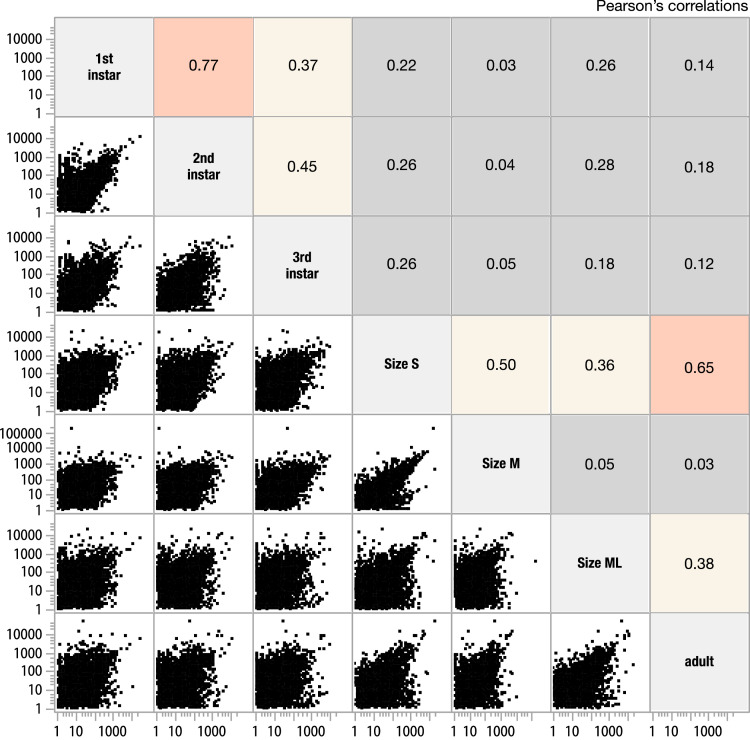
Correlation of the expression levels in TPM of all seven samples. Correlation is higher among the first three instars and among later stages (S, M, ML, and adult). These correlations suggest specific gene expression in the early life stages.

Reference transcriptome assembly was constructed from subsampled reads from all samples to achieve maximum coverage. Since we have previously seen a small but certain level of cross-contaminations with multiplexed sequencing, we have eliminated assembled transcripts that are less than 1 TPM. However, even after this screening, as the summary statistics shows in Table 2, The assembly contains a large number of assembled contigs, with Complete + Partial BUSCO score of 99.44%, indicating that this is a very comprehensive assembly. This assembly file in FASTA format is available as Supplementary File 1. This reference was used to quantify and visualize the expression levels of the seven RNA-Seq datasets. Tab-delimited text data of all expression values of all genes is attached as Supplementary File 2. The expression profiles shown as heatmap clustering (Fig. 1) shows reproducible expression in neighboring life stages, and specific gene expression patterns in early and late developmental stages. Correlation among the samples are also shown in Fig. 2, where strong correlation is seen among 1st three instars and among later stages (S, M, ML, and adult samples).

**Table 2:** Summary of transcriptome assembly statistics.

Scaffold number	126,310
Total scaffold length	112,679,075
Average scaffold length	892
Longest scaffold	25,566
Shortest scaffold length	176
N50 length (contig #)	1944 (#15,969)
N90 length (contig#)	306 (#79,565)
BUSCO Completeness (%)	96.90
BUSCO Complete + Partial (%)	99.44

## 2 Experimental Design, Materials and Methods

Sample preservation, RNA extraction, sequencing and assembly were conducted using methods previously described for spiders [1], with some modifications. Briefly, a whole body of the single specimen of each of the developmental stages of *G. bimaculatus* was snap-frozen with liquid nitrogen and stored at −80 °C, and RNA was extracted using TRIzol reagent (Thermo Scientific) after homogenization with a metal cone using the Multi Beads Shocker (Yasui Kikai). RNA was further purified using RNeasy Plus Mini Kit (Qiagen). Purified total RNA was quantified with a NanoDrop 2000 (Thermo Scientific) and a Qubit Broad Range (BR) RNA assay (Life Technologies). The integrity of RNA was estimated by electrophoresis using TapeStation 2200 with RNA Screen Tape (Agilent Technologies). The sequence library was prepared using NEB Next Ultra II RNA Library Prep Kit for Illumina (NEB) according to manufacturer's protocol, and then was sequenced on a NextSeq 500 (Illumina) with 300 cycles of high-output mode as paired-end reads. Sequences were base called and demultiplexed, and adaptor sequences were removed with bcls2fastq v.2 software (Illumina).

To generate a reference transcriptome assembly, duplicate reads were first removed using CD-HIT, and 6 M paired reads were subsampled from the seven samples for computational efficiency. Transcriptome assembly was then performed on the subsampled data using Bridger software with default parameters [2]. Expression levels of transcripts were quantified with Kallisto v.0.44 [3] using the assembled transcriptome, firstly using the subsampled data, and contigs with less than 1 TPM (transcript per million) expression were removed to eliminate possible cross-contaminations during multiplexed sequencing. Assembly completeness was assessed using BUSCO v.3 [4] with the Arthropoda dataset through the gVolante server [5]. Using the refined reference, expression levels of all conditions were calculated with Kallisto. Statistical analyses were performed using JMP software v.14.1 (SAS Institute).

## Ethics Statement

All experiments were conducted following the Japanese law and guidelines from The Science Council of Japan, as well as the Ministry of Education, Culture, Sports, Science and Technology (MEXT) of Japan.

## CRediT authorship contribution statement

**Nobuaki Kono:** Methodology, Validation, Formal analysis, Investigation, Data curation, Writing – review & editing. **Hiroyuki Nakamura:** Resources, Writing – review & editing. **Rintaro Ohtoshi:** Resources, Writing – review & editing. **Kazuharu Arakawa:** Conceptualization, Methodology, Validation, Data curation, Visualization, Project administration, Funding acquisition, Writing – original draft.

## Declaration of Competing Interest

Hiroyuki Nakamura and Rintaro Ohtoshi are employees of Spiber Inc., a company selling protein materials. However, Spiber Inc. had no role in study design and formal analysis.

## Appendix Supplementary materials

Supplementary File 1: Reference transcriptome assembly of *G. bimaculatus* (FASTA format). Image, application 1

Supplementary File 2: Tab-delimited text file of expression values (TPM) of all transcripts. Image, application 2

## References

[1] Kono N., Nakamura H., Ito Y., Tomita M., Arakawa K. (2016). Evaluation of the impact of RNA preservation methods of spiders for de novo transcriptome assembly. Mol. Ecol. Resour..

[2] Chang Z., Li G., Liu J., Zhang Y., Ashby C., Liu D. (2015). Bridger: a new framework for de novo transcriptome assembly using RNA-seq data. Genome Biol..

[3] Bray N.L., Pimentel H., Melsted P., Pachter L. (2016). Near-optimal probabilistic RNA-seq quantification. Nat. Biotechnol..

[4] Seppey M., Manni M., Zdobnov E.M. (2019). BUSCO: assessing genome assembly and annotation completeness. Methods Mol. Biol..

[5] Nishimura O., Hara Y., Kuraku S. (2017). gVolante for standardizing completeness assessment of genome and transcriptome assemblies. Bioinformatics.

